# Automatic approach for B-lines detection in lung ultrasound images using You Only Look Once algorithm

**DOI:** 10.1007/s40477-025-01077-w

**Published:** 2025-09-11

**Authors:** Alberto Bottino, Chiara Botrugno, Ernesto Casciaro, Francesco Conversano, Aimé Lay-Ekuakille, Fiorella Anna Lombardi, Rocco Morello, Paola Pisani, Luigi Vetrugno, Sergio Casciaro

**Affiliations:** 1 Department of Innovation Engineering, University of Salento, Lecce, Italy; 2 National Research Council – Institute of Clinical Physiology, Lecce, Italy; 3 Micro Nano Sensor Group Polytechnic University of Bari, Bari, Italy; 4 Department of Medical, Oral and Biotechnological Sciences, University of Chieti-Pescara, Chieti, Italy; 5 Department of Anaesthesiology, Critical Care Medicine and Emergency, SS. Annunziata Hospital, Via Dei Vestini, 66100 Chieti, Italy; 6 Consiglio Nazionale Delle Ricerche, Istituto Di Fisiologia Clinica (CNR-IFC), c/o Campus Universitario Ecotekne, via per Monteroni, 73100 Lecce, Italy

**Keywords:** Lung ultrasound, You Only Look Once, Detection algorithm, B-lines

## Abstract

**Purpose:**

B-lines are among the key artifact signs observed in Lung Ultrasound (LUS), playing a critical role in differentiating pulmonary diseases and assessing overall lung condition. However, their accurate detection and quantification can be time-consuming and technically challenging, especially for less experienced operators. This study aims to evaluate the performance of a YOLO (You Only Look Once)–based algorithm for the automated detection of B-lines, offering a novel tool to support clinical decision-making. The proposed approach is designed to improve the efficiency and consistency of LUS interpretation, particularly for non-expert practitioners, and to enhance its utility in guiding respiratory management.

**Methods:**

In this observational agreement study, 644 images from both anonymized internal and clinical online database were evaluated. After a quality selection step, 386 images remained available for analysis from 46 patients. Ground truth was established by blinded expert sonographer identifying B-lines within rectangular Region Of Interest (ROI) on each frame. Algorithm performances were assessed through Precision, Recall and F1 Score, whereas to quantify the agreement between the YOLO-based algorithm and the expert operator, weighted kappa (kw) statistics were employed.

**Results:**

The algorithm achieved a precision of 0.92 (95% CI 0.89–0.94), recall of 0.81 (95% CI 0.77–0.85), and F1-score of 0.86 (95% CI 0.83–0.88). The weighted kappa was 0.68 (95% CI 0.64–0.72), indicating substantial agreement algorithm and expert annotations.

**Conclusions:**

The proposed algorithm has demonstrated its potential to significantly enhance diagnostic support by accurately detecting B-lines in LUS images.

**Supplementary Information:**

The online version contains supplementary material available at 10.1007/s40477-025-01077-w.

## Introduction

Lung ultrasound (LUS) is a safe and effective tool for diagnosing patients with acute respiratory failure in adult, pediatric, and neonatal intensive care or emergencies [1, 2]. It acts as a first-level exam for children and adults, offering real-time visualization of key lung artifacts such as A-lines and B-lines, lung sliding, as well as objective pathological findings such as atelectasis, consolidation, pleural effusion, and air bronchogram, thereby providing immediate insights into the patient’s condition [3–6].

Under normal lung aeration, A-lines and lung sliding are typically present, whereas B-lines serve as critical diagnostic artifacts. The presence of three or four B-lines between two ribs is associated with thickened subpleural interlobular septa; five B-lines or more (creating a white lung pattern) correlates with CT ground-glass opacities (GGO), indicating severe interstitial syndrome or de-aeration [7].

Despite its diagnostic utility, the low specificity of sonographic interstitial syndromedoes not allow the operator to classify diseases that can cause B-lines. Nevertheless, in the clinical context, B-lines helped differentiate cardiogenic pulmonary oedema from exacerbation of chronic obstructive pulmonary disease (COPD) with a sensitivity of 100% and a specificity of 92% [8]. Furthermore, studies also demonstrate that the number of B-lines inversely correlates with pulmonary function tests (PFT) and (Diffusing Capacity for Carbon Monoxide) DLCO, suggesting that B-lines hold both diagnostic value and pathophysiological significance, which could be crucial for ongoing monitoring. The SARS-CoV-2 pandemic has further highlighted B-lines’ significance in diagnosing interstitial viral pneumonia. Likewise, using the LUS score for both a semi-quantitative assessment of the lung surface and to evaluate the underlying disease extension can add additional value to better choose the proper support for the patient, for example, non-invasive versus invasive mechanical ventilation support or to predict the risk of orotracheal intubation and in-hospital mortality. Moreover, LUS could also be a valid support for evaluating lung recruitability, monitoring and verifying the effectiveness of the recruitment maneuvers, and identifying any early complications [9]. Nowadays, the LUS score can be calculated in two ways: by an expert certificate LUS sonographer or with AI-based algorithms. In the first case, the insufficient number of tutors resulted in 40% of LUS practitioners performing the procedure without completing a training course and 76.7% without certification in the ICU setting [10]. In the second case, different AI-based algorithms, such as ResNet, DenseNet, and YOLO can help non-certificate doctors work at the bedside or at a distance. For these reasons in this study, we aim to develop a YOLO-based model to detect and count B-lines in LUS images, validate its performance against expert annotations, and explore pathways for real-time clinical integration into point-of-care ultrasound devices, offering a novel approach to support LUS evaluation procedure and its potential applications.

Clinically speaking, the YOLO-based algorithm for B-lines counting can be used in different clinical settings, from emergency departments to intensive care for adults, pediatrics, and neonatal patients. Lichtenstein et al.’s most impactful article focused on the “Bedside Lung Ultrasound in Emergency” (BLUE) protocol, which showed that B-lines helped differentiate cardiogenic pulmonary oedema from exacerbation of chronic obstructive pulmonary disease (COPD) [8]. After some years, The European Association of Cardiovascular Imaging in 2013 suggested that the absence of multiple bilateral B-lines excludes cardiogenic pulmonary oedema with a negative predictive value close to 100% [11]. In contrast, B-lines were significantly correlated with new-onset acute congestive heart failure when their number was ≥ 15 per scan; this cutoff could be considered for a quick and reliable assessment of decompensated heart failure (HF) [12]. Differentiating these two scenarios by accurately and rapidly counting B-lines with the YOLO algorithm could be easy: for example, the algorithm could reliably track disease progression during acute and recovery phases but may struggle during the florid state (e.g., white lung or multiple B-lines overlapping). In cardiogenic pulmonary oedema, characterized by a thin pleura and a B-profile extending from the base to the apex, LUS achieves 97% sensitivity and 95% specificity [13]. Moreover, studying the anterolateral zones of the thorax, considering the posterior zone suffers from fluid gravity in the distribution zone, could help as the regional LUS score sometimes works better than the global LUS score.

Another potential application of the YOLO algorithm could be to evaluate the patient’s lung fluid tolerance: this could be fundamental, considering that fluid administration in the ICU is the cornerstone of treating critically ill patients. For example, after the fluid challenge, the lung could be considered”wet” (B-profile) when B-lines increase by 8%, with a heterogeneous distribution in the lung and aeration loss as B-lines are directly related to extra vascular lung water (EVLW) and higher mortality [14, 29]. Furthermore, the interstitial syndrome also presents focal B-profile or diffuse B-lines in Acute Respiratory Distress Syndrome (ARDS) [15], requiring different treatment positions: low Positive End-Expiratory Pressure (PEEP) and prone position in the first case and increased PEEP in the second case. The distinction between global and regional LUS scores is important for the diagnosis in the early phase: since lung recruitment involves particularly poorly aerated areas rather than consolidated regions, a LUS score allows clinicians to quantify the relative share between these two patterns, helping to set appropriate mechanical ventilation parameters, balance alveolar recruitment and reduce the risk of lung overinflation. Thus, the YOLO algorithm, by detecting and counting B-lines, could be useful also in this situation, reducing the number of diagnostic tests that use ionizing radiation. For example, as stated in 2016 by the European Society of Cardiology Guidelines [16], for the diagnosis and treatment of acute and chronic heart failure, chest X-ray use is limited to the diagnostic work-up of patients with suspected HF and in identifying an alternative pulmonary explanation for patient’s symptoms and signs.

For these reasons, the primary objective of this study (i.e., the automated detection and quantification of individual B-lines in LUS images) can be beneficial in different applications of LUS and, more in general, in assessing lung condition. At this time, although accurate and automated B-line quantification can support clinical classification, we did not extend the algorithm to binary clinical classification (normal vs abnormal lung) or differential diagnosis in this work; however, frameworks for such applications have been discussed in prior studies [17].

## Methods

This observational agreement study was conducted at the Hospital, Vito Fazzi, Lecce, Italy, after approval of Ethic Committee ID protocol #50518//21. We analyzed an extensive anonymized internal and clinical online database from Butterfly and Grepmed (butterflynetwork.com and grepmed.com). The inclusion study criteria were adult hospitalized patients with satisfactory ultrasound windows willing to participate in the investigation. Exclusion criteria were the inability to obtain informed patient consent, knowledge of lung fibrosis, and/or previous cardiothoracic surgery. We only utilized video clips of these patients that respected the European General Data Protection Regulation 2016/679 (GDPR).

The acquisitions were performed using a portable ultrasound device named SensUS Lung (Amolab Srl, Lecce, Italy; https://www.amolab.it/en/homepage/). This device comprises a tablet PC with a 3.5 MHz convex transducer. A cutting-edge acquisition technique was implemented: LUS frames were obtained using the 14-zone method, comprising 7 zones on each side (left and right) [18]. In total, 24 frames per zone were recorded, with the probe scanning each zone in both longitudinal and transverse directions, capturing 12 frames per direction. For each zone, the first frame, (usually the most representative, as it coincides with the moment when the acquisition begins) out of the 24 acquired was selected. Three variable parameters were considered during each acquisition: depth, focus, and gain. Depth (ranging from 90 to 210 mm) was set according to the patient’s BMI; the focus was set as close as possible to the pleural line level, and the operator selected a gain value to obtain the best visualization of the pleura. Low-resolution frames, where the pleura was not visible, or low-contrast images were discarded. The obtained images were saved as Joint Photographic Experts Group (JPEG), a lossless compression image format, and grouped. To avoid overfitting and ensure robust model performance, a fivefold cross-validation approach was implemented. This method divides the dataset into five subsets of equal size. In each iteration, four folds were used for training and fine-tuning the network parameters, while the remaining fold was used for validation and testing. This process was repeated five times, with each fold being used as the test set exactly once, ensuring all images were evaluated unbiasedly. The expert sonographer communicated with the study’s Principal Investigator in case of any difficulties or problems with the video, and a third expert was involved in case of discrepancies. The expert LUS sonographer was blinded to the YOLO detection results and selected the B-lines on each frame through a rectangular Region Of Interest (ROI). Given an image, the number of this rectangular ROI drawn by the operator also corresponded to the number of B-lines on that image. Due to the subjectivity in identifying overlapping B-lines, a standardized visual rule was used: if multiple vertical artifacts shared an origin and could not be visually separated at the pleural line, they were considered coalescent and counted as a single B-line; otherwise, they were marked with separate ROIs. This annotation protocol served as the gold standard.

**Fig. 1:**
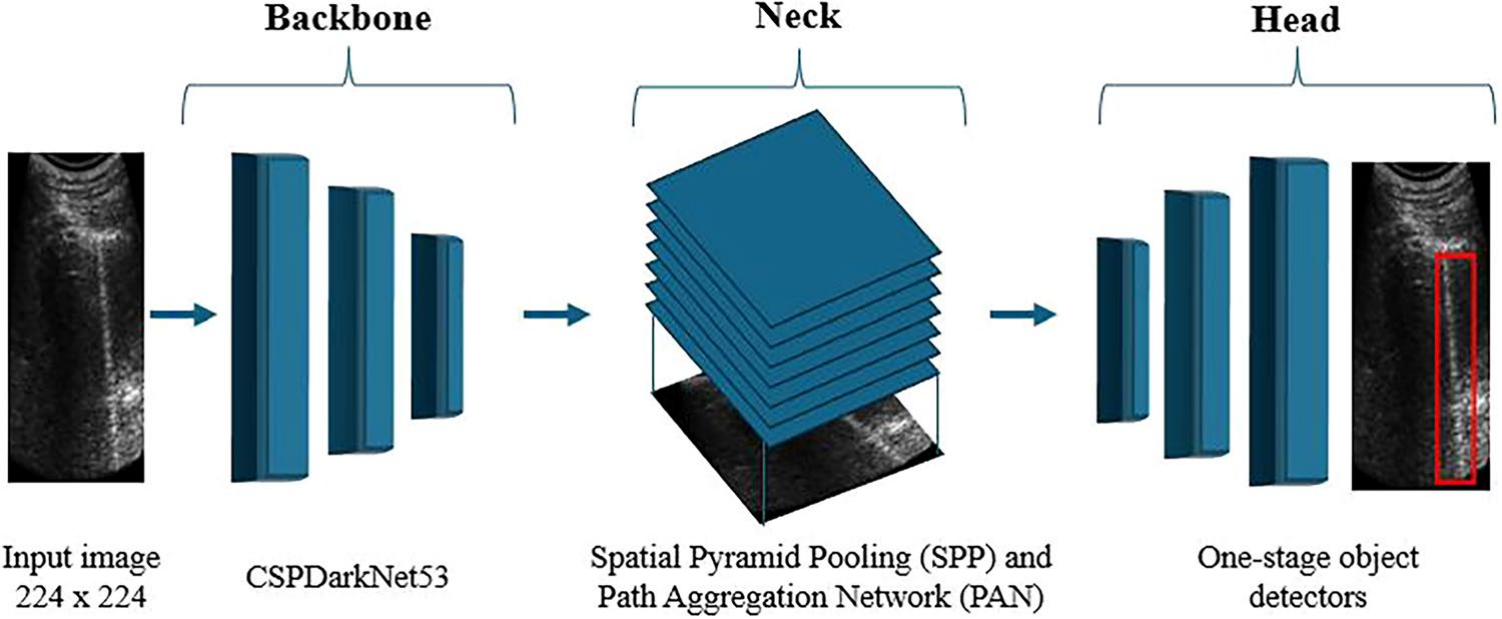
YOLO architecture The model comprises three main components: (1) the backbone, CSPDarkNet53, pre-trained on the COCO dataset for feature extraction; (2) the neck, integrating a spatial pyramid pooling (SPP) module and a path aggregation network (PAN) for enhanced feature representation; and (3) the head, designed as a one-stage object detector for efficient object localization and classification

AI-based object detection algorithm YOLO is a detection algorithm based on Convolutional Neural Networks (CNNs) that has assumed a leading role in all fields of object recognition in recent years [19–21]. Through its innovative architecture, it combines speed and accuracy. The implementation and training of the YOLO-based object detection algorithm were carried out using MATLAB^®^ 2023a (The MathWorks, Inc., Natick, Massachusetts, USA), with all test and code development performed in this environment. The architecture of the YOLO algorithm used in this study was made of:

- 1. A backbone for the feature extraction (CSPDarkNet53 trained on COCO data sets).
- 2. A neck comprises a spatial pyramid pooling (SPP) module and a path aggregation network (PAN).
- 3. The head by one-stage object detectors (Fig. 1).

Starting from a learning rate of 3e-4 and fixing a gradient decay factor to 0.9, a max epoch limit of 50 was imposed. Adam optimizer was used for parameter optimization.

**Fig. 2:**
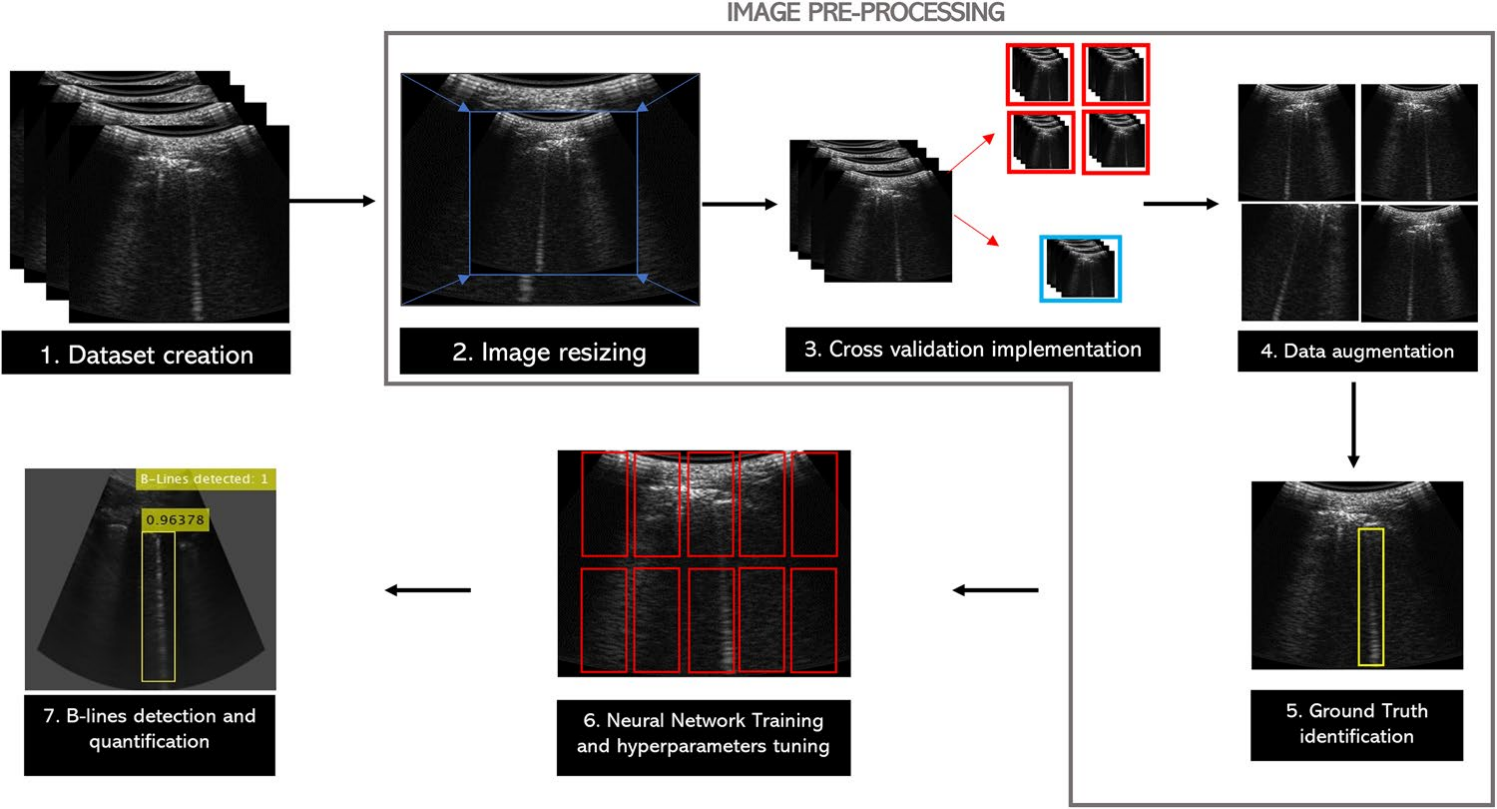
Algorithm workflow Steps include: (1) dataset creation; (2) resizing to 224 × 224; (3) fivefold cross-validation; (4) data augmentation; (5) expert annotation; (6) neural network training with optimized hyperparameters; and (7) detection and quantification of B-lines. The output of step 7 provides both ROI localization for each B-line and a confidence score, enabling quantitative scoring for each scan zone

The algorithm for processing the LUS images involved the following steps (Fig. 2): 1) Datasets creation; 2) Image resizing; 3) Cross-validation implementation; 4) Data Augmentation; 5) Ground Truth identification; 6) Neural Network Training and hyperparameter tuning; 7) B-lines detection and quantification (Additional file 1).

**Fig. 3:**
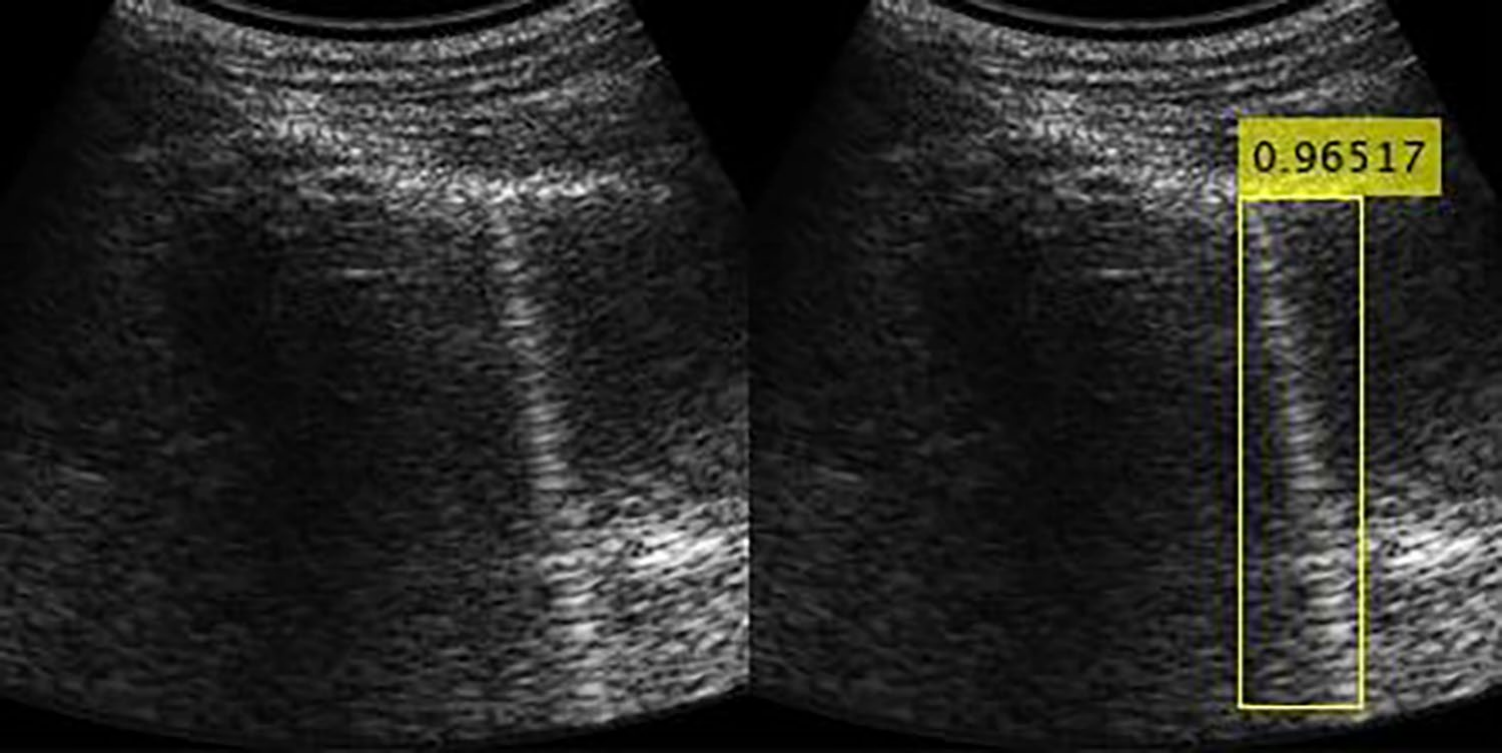
Example of algorithm output Left: original LUS frame. Right: detected B-lines (white boxes) with overlaid confidence scores. This output illustrates the model’s ability to localize individual B-lines for subsequent lung aeration scoring or threshold-based clinical decision support

The final output consists of the identified b-lines with the rectangular ROIs accompanied by a score which is the probability that the identified object is indeed a B-line (Fig. 3).

The first end-point of this study was the agreement between the YOLO-based detection algorithm and the expert sonographer. The second end-point was the good balance between precision and speed reached by the algorithm.

To quantify the agreement between the YOLO-based algorithm and the expert operator, weighted kappa (k_w_) statistics were employed as weighting schemes considering the closeness of agreement between categories. In this study, the Fleiss-Cohen weighting system was used based on inverse-square spacing. In particular, k_w_ ranges from −1 to 1, where 1 indicates perfect agreement, 0 represents chance-level agreement, and values below 0 suggest worse-than-chance agreement. Interpretation thresholds classify agreement as slight (0.00–0.20), fair (0.21–0.40), moderate (0.41–0.60), substantial (0.61–0.80), or almost perfect (0.81–1.00) [22]. This approach, also known as quadratic weights, since it is proportional to the square of the deviation of separate ratings, prioritizes deviations that are closer to the true values, providing a robust measure of agreement for ordinal data. The algorithm’s performance was evaluated using precision and recall. Defined correctly recognized B-lines as True Positive (TP) and incorrectly predicted B-line as False Positive (FP), the precision was defined as:

1 $$Precision=\frac{TP}{(TP+FP)}$$

whereas the recall was:

2 $$Recall= \frac{TP}{(TP+FN)}$$

where FN represented the number of false negatives (i.e., how many B-lines the algorithm missed).

In particular, Precision is analogous to the Positive Predictive Value (PPV) as it reflects the proportion of correctly detected B-lines among all detections made by the algorithm. In contrast, recall indicates the sensitivity of the model by quantifying the proportion of actual B-lines that are correctly identified. Clinically, a high precision minimizes false positives (thus avoiding unnecessary interventions), while a high recall is crucial for ensuring that subtle or faint B-lines, which may signal early or severe pathology, are not overlooked.

In addition, the F-1 score, a weighted mean of precision and recall, has been evaluated as follows:

3 $$F1 Score= \frac{(2 \times Precision\times Recall)}{(Precision+Recall)}$$

Since True Negatives (TN) could not be well-defined from a physical point of view, but as it was necessary to evaluate k_w_, it was estimated as the total number of images without False Positives.

Both the statistical analysis and the algorithm performance evaluation were performed using MATLAB^®^ tools.

## Results

A total of 644 frames were evaluated. Images in which the pleura line was not visible or with a high image-to-noise ratio were eliminated. After this quality selection, 386 images remained available for analysis from 46 patients. Subsequently, the first step of image pre-processing involved resizing the images to 224 × 224, which is the required input dimension for the model. A fivefold cross-validation was implemented to evaluate the neural network’s performance and reduce the risk of overfitting. In each of the five iterations, 80% of the images (309) were used or training. In contrast, the remaining 20% (77 images) were used for validation and testing, ensuring every image was tested simultaneously. Separately, images in the dataset served as part of the validation set exactly once across the five iterations. In total, 435 B-lines were identified across the five validation sets (an average of 87 per fold). Of these, 352 instances were accurately identified as true positives (TP = 352), while 83 instances were not detected by the model, resulting in false negatives (FN = 83). Additionally, the model produced 31 false positives (FP = 31) across all the validation folds. The aggregated performance metrics from the five iterations demonstrated an overall Precision rate of 0.92 (95% Confidence Interval (CI) 0.89–0.94), whereas the recall rate, reflecting the proportion of actual B-lines accurately detected by the model, stood at 0.81 (95% CI 0.77–0.85). This balance between precision and recall led to an F1-score of 0.86 (95% CI 0.83–0.88).

In addition, a Fleiss-Cohen weighted kappa kw equal to 0.68 (95% CI 0.64–0.72) was found.

## Discussion

The achieved performance suggests that the algorithm prioritizes precision over recall, minimizing false positives but missing some B-lines. This high precision is analogous to a high positive predictive value, meaning that when the algorithm indicates the presence of a B-line, it is highly reliable. In other words, as B-lines are pre-radiological and preclinical signs, the system can be considered “conservative”, accurately distinguishing most identified B-lines but with a slight loss in sensitivity, meaning not all B-lines were recognized when they are too much. Another important application of the developed YOLO algorithm could be predicting the success or failure from mechanical ventilation during the weaning phase. For example, weaning-induced pulmonary oedema (WIPO), manifests as increased B-lines after discontinuing positive pressure from the ventilator. In this contest, the YOLO algorithm, if used for prognostic purposes, is mainly helpful for identifying low-risk cases with their high negative predictive value. In particular, precision values reported in literature typically range from 0.85 to 0.95, with high-performing models achieving a precision above 0.90. Thus, the precision of 0.92 obtained here suggests that YOLO-based algorithm better minimizes false positives respect to other AI-based algorithms for B-lines detection, such as ExoLungAI (which, however, obtained a sensibility of 0.84) [23]. For instance, in intensive care or emergency room settings, where rapid decision-making is needed, minimizing false positives is essential to avoid unnecessary treatments. Moreover, compared to findings in the literature, our algorithm was trained and validated on a large cohort of patients. For instance, Karakus et al. achieved 87% accuracy in detecting B-lines but tested their algorithm on nine patients [24]. Similarly, Russell et al. [25] and Camacho et al. [26] (who reported 88% agreement between their algorithms and expert assessments for B-line detection), enrolled for their studies 29 and 28 patients, respectively. To our knowledge, no data exist about pediatric and neonatology settings where LUS has been used to evaluate the need for surfactant, bronchopulmonary dysplasia development, bronchiolitis, and mechanical ventilation, but this could be another critical setting in which the YOLO algorithm could work. However, according to the Landis and Kock benchmark scale, for adult setting the computed Fleiss-Cohen weighted kappa of 0.68 showed substantial agreement between the algorithm and expert annotations, confirming its reliability tool for potential assisting clinicians in diagnosing lung diseases. Quantitative LUS thresholds specific to clinical conditions have been proposed, though limitations must be acknowledged. The “grid-based” detection architecture can still present challenges, especially when objects are positioned close to each other, like in the case of multiple clustered B-lines. However, the algorithm’s performance metrics align well with findings in recent studies focusing on B-lines detection in lung ultrasound: in literature, recall values for B-lines detection obtained with other algorithms commonly fall between 0.70 and 0.85 [27, 28], reflecting challenges in detecting subtle or faint B-lines artifacts in suboptimal imaging conditions. Excluding low-quality images was necessary to ensure that the data analyzed met a minimum quality standard, allowing for a more accurate and reliable interpretation of pleural features. While this may have led to the omission of more challenging cases, this reflects real-world practices where operators adjust parameters or repeat scans to enhance image quality; indeed, frames where the pleura is entirely invisible or heavily obscured by noise, are not informative. Improving recall, particularly for faint B-lines obscured by noise, remains a key focus for algorithm refinement.

Despite these challenges, YOLO could significantly benefit clinical practice: by automatically detecting and counting B-lines, it can accelerate the computation of standardized LUS scores, potentially reducing inter-operator variability and reporting time at the bedside. Although our current evaluation was performed on saved video frames—with low per-frame inference times on (hundreds of milliseconds)—the inherent efficiency of YOLO architectures makes real-time video processing entirely feasible. Future development will focus on integrating the model into ultrasound consoles or portable tablets, overlaying bounding boxes and count summaries directly on the operator’s display. This capability would furnish clinicians with instantaneous decision support, flagging high B-line counts or rapid changes in aeration patterns during dynamic maneuvers such as recruitment testing or weaning trials.

## Conclusions

The proposed YOLO-based algorithm has demonstrated its potential to significantly enhance diagnostic support by accurately detecting B-lines in LUS images. These results collectively suggest that the YOLO-based model offers a strong balance between precision and recall, achieving performance metrics comparable to or exceeding those reported in the literature. The emergence of automated detection technologies holds significant promise for improving diagnostic accuracy and accessibility. However, ongoing research and collaboration between clinicians and technology developers are crucial. As we move forward, a balanced approach that integrates AI-driven innovations with established clinical expertise will be essential for optimizing patient outcomes in respiratory medicine.

## Supplementary Information

Below is the link to the electronic supplementary material.

Supplementary file1 A more detailed description of the algorithm applied for LUS images processing. (DOCX 19 KB)

## Data Availability

For this study, both internal and clinical online databases have been used. Online database has been taken from Butterfly at butterflynetwork.com and Grepmed at grepmed.com. Internal data available on request due to privacy/ethical restrictions.
