# Supplementary material for: Automatic approach for B-lines detection in lung ultrasound images using You Only Look Once algorithm
Source: J Ultrasound. 2025 Sep 11;28(4):985–92. doi: 10.1007/s40477-025-01077-w (PMC12675886; doi:10.1007/s40477-025-01077-w)
Supplement: Supplementary file 1 — Supplementary file1 A more detailed description of the algorithm applied for LUS images processing. (DOCX 19 KB) [file 40477_2025_1077_MOESM1_ESM.docx]

**ADDITIONAL FILE 1**

The algorithm applied for LUS images processing for B-lines detection involved the following steps:
1) Datasets creation. All 644 images have been evaluated to discard low-quality ones. Images where the pleura line was not visible or with a high image-to-noise ratio have been eliminated. A total of 386 images remained after this quality selection.

2) Image resizing. The first step of image pre-processing involved resizing the images to 224x224, the required input dimensions for the model.

3) Cross-validation implementation. The remaining dataset of 386 images was used in a 5-fold cross-validation framework, where the images were randomly divided into five equally sized folds. n each of the five iterations, 80% of the images (309) were used for training, while the remaining 20% (77 images) were used for validation and testing, ensuring every image was tested exactly once across the process.

4) Data Augmentation. Lightly rotation (± 10°), translation, and brightness variation techniques have been applied to each i-th training set images to increase the variety and the number of clinical records to give as input to YOLO Neural Networks.

5) Ground Truth identification. An experienced operator analyzed each image and identified the B-lines within a rectangular ROI.

6) Neural Network Training and hyperparameter tuning. The hyperparameters of neural networks, such as learning rate, number of hidden layers, and batch size, have been optimized to achieve a good trade-off between computational time and output accuracy.

7) B-lines detection and quantification. The detected B-lines were highlighted with a rectangular ROI, providing a clear visual representation of the confidence score, enabling quantitative scoring for each scan zone.
